# Soluble fibrinogen‐like protein 2 ameliorates acute rejection of liver transplantation in rat via inducing Kupffer cells M2 polarization

**DOI:** 10.1002/cam4.1528

**Published:** 2018-05-10

**Authors:** Guangrui Pan, Zhengfei Zhao, Chengyong Tang, Liuyue Ding, Zhongtang Li, Daofeng Zheng, Liang Zong, Zhongjun Wu

**Affiliations:** ^1^ Department of Hepatobiliary Surgery The First Affiliated Hospital of Chongqing Medical University Chongqing China; ^2^ Department of Clinical Pharmacology The First Affiliated Hospital of Chongqing Medical University Chongqing China; ^3^ Medical Research Center Su Bei People's Hospital of Jiangsu Province Yangzhou University Yangzhou China; ^4^ Department of Surgery Su Bei People's Hospital of Jiangsu Province Yangzhou University Yangzhou China

**Keywords:** acute rejection, Kupffer cells, M2 polarization, orthotopic liver transplantation, soluble fibrinogen‐like protein 2

## Abstract

Soluble fibrinogen‐like protein 2 (sFGL2) could ameliorate acute rejection (AR) in rat cardiac transplantation. However, the role of sFGL2 in AR of liver transplantation has not been addressed. In this study, we found that FGL2 was upregulated in rat orthotropic liver transplantation (OLT) models of tolerance and positive correlation with the frequency of M2 Kupffer cells (KCs). Gain‐of‐function experiments in vitro showed that sFGL2 promoted the secretion of anti‐inflammatory cytokines (IL‐10, TGF‐β) and the expression of CD206, and inhibited the activities of STAT1 and NF‐κB signaling pathway. Consistently, in vivo assays showed that adeno‐associated virus‐mediated FGL2 (AAV‐FGL2) transfer to recipients could ameliorate AR of rat OLT and induce KCs M2 polarization in allografts. Notably, we found that the recipients receiving transferred KCs from AAV‐FGL2‐treated allograft showed alleviated AR. Taken together, we revealed that sFGL2 ameliorated AR by inducing KCs M2 polarization.

## 1 INTRODUCTION

AR is the major barrier to the survival of recipients receiving liver transplantation despite the application of immunosuppressive agents.^1^ New therapeutics are to make up for the deficiency in specificity and selectivity of immunosuppressive agents.^2^ It has been reported that the innate immune system activation to inflammation and tissue injury initiates and amplifies the adaptive response which is critical for AR.^3^, ^4^ Therefore, uncovering key molecules regulating innate immunity is important to develop new anti‐rejection agents.

KCs, liver‐resident macrophages, are the key modulator of innate immune system in liver.^5^ Macrophages can shift their functional polarization status between M1 and M2 due to their plasticity.^6^, ^7^ M1 macrophages are induced by LPS and IFN‐γ, secreting pro‐inflammatory cytokines, while M2 macrophages induced by IL‐4, secreting anti‐inflammatory cytokines.^8^ In the early stage of rat liver ischemia‐reperfusion injury (IRI), KCs are activated and release pro‐inflammatory cytokines such as IL‐1, IL‐6, and TNF‐α.^9^, ^10^ In contrast, an M2 KC‐dominant response ameliorates IRI in miR‐155 deficiency mice.^11^ It has been shown that M1 and M2 macrophages regulate type 1 T helper cell (Th1) and type 2 T helper cell (Th2) responses, respectively.^12^ Activated KCs promote intra‐hepatic Th1 cell infiltration in a necro‐inflammation mouse model.^13^ On the other hand, KCs derived from accepted liver allograft inhibit AR of rat OLT by inducing T cells apoptosis and promoting Th2 cell differentiation.^14^ These findings suggest that the role of KCs is associated with their polarization state and the increase in M2 KCs could ameliorate AR of OLT.

FGL2, a member of fibrinogen family, has 2 protein forms, membrane‐bound form (mFGL2) with coagulation activity and secreted form (sFGL2) with immunosuppressive properties.^15^, ^16^ FGL2 is involved in regulating progressions of many diseases.^17^, ^18^, ^19^, ^20^, ^21^, ^22^, ^23^, ^24^ FGL2 deletion in Tregs contributes to the development of autoimmune glomerulonephritis in mice.^21^ FGL2 is upregulated in tolerant cardiac and liver allografts in mice, and over‐expression of FGL2 could inhibit rat cardiac allograft rejection.^22^, ^23^ Recent study shows that FGL2 promotes glioma growth by expanding tumor‐associate macrophages via FcγRIIB pathway.^24^ These suggest that FGL2 is involved in the regulation of macrophages function and transplant tolerance. However, little is known about the effects of sFGL2 on AR of OLT by regulating KCs.

In this study, we found that FGL2 expression and M2 KCs were increased in tolerance group of rat OLT. We also found that sFGL2 could induce KCs M2 polarization and inhibit STAT1 and NF‐κB signaling pathway in vitro. Importantly, sFGL2 could inhibit AR of rat OLT via inducing KCs M2 polarization in vivo.

## 2 MATERIAL AND METHODS

### 2.1 Animals and liver transplantation

Lewis and Brown Norway (BN) rats were obtained from Animal Research Center of Chongqing Medical University (Chongqing, China) and maintained in a specific pathogen free (SPF) environment. The AR (Lewis to BN) and tolerance model (BN to Lewis) of OLT were performed according to the two‐cuff method from Kamada.^25^ The ischemic time was between 13 and 18 minutes. There were 6‐8 recipients weighing 230‐280 g in each group.

### 2.2 Isolation of KCs and cell Culture

KCs were isolated and purified from liver tissue using modified methods^26^ (Figure S1). KCs were cultured into six‐well plate at a density of 1 × 10^6^/well in media, containing RPMI 1640 (Hyclone, USA) with 10% Fetal Bovine Serum (Hyclone, USA) and 1% streptomycin and penicillin (Sigma, USA), at 37°C under an atmosphere of 5% CO_2_.

### 2.3 Treatment of KCs

We used r‐FGL2 (Cloud‐clone, USA) in vitro experiment. KCs were co‐stimulated with or without 1 μg/mL LPS and 20 ng/mL IFN‐γ (LPS/IFN‐γ) (Sigma, USA) 27 in the presence of r‐FGL2 (4 μg/mL) for 24 hours (Figure S2). KCs cultured in normal media were set as control group.

### 2.4 Flow cytometry analysis

KCs were collected and stained with PE‐anti‐CD206, FITC‐anti‐F4/80, or specific isotype antibodies (eBioscience, USA). BD FACS Calibur flow cytometer was used, and data were processed with CytExpert software.

### 2.5 Cytokine assay

KCs culture supernatants were collected 24 hours after treatment and rat serums were collected on day 7 after OLT. The levels of IL‐12, TNF‐a, IL‐10, TGF‐β (4A Biotech, China), and sFGL2 (Cloud Clone, USA) were examined according to the manufacturer's instructions of ELISA Kits.

### 2.6 Quantitative real‐time RT‐PCR (qRT‐PCR)

Total RNA was extracted using TRIzol reagent (Takara, Japan) and used as a template for reverse transcription into cDNA with Reverse Transcript Reagents kit (Takara, Japan). The analysis was performed in a Real‐Time Detection System (ABI 7500, Thermo Fisher Scientific) following the manufacturer's instructions for the SYBR Prime Script RT‐PCR Kit (Bio‐RAD, USA). The primers were listed in Table 1.

**Table 1:** Primers used in mRNA expression analysis

Gene name	Primer sequence	
	Foward	Reverse
FGL2	CAAGAACACAACCAGCCAAATCC	CCCAGCCAAAATTCTCGTTCAA
IL‐12	CAGAAGCTAACCATCTCCTGGTTTG	TCCGGAGTAATTTGGTGCTTCACAC
TNF‐α	CCGATTTGCCACTTCATACCA	TAGGGCAAGGGCTCTTGATG
IL‐10	TGCTATGTTGCCTGCTCTTACTG	TCAAATGCTCCTTGATTTCTGG
TGF‐β	TTGCTTCAGCTCCACAGAGA	TGGTTGTAGAGGGCAAGGAC
Arg‐1	CCGCAGCATTAAGGAAAGC	CCCGTGGTCTCTCACATTG
GAPDH	GGTGGACCTCATGGCCTACA	CTCTCTTGCTCTCAGTATCCTTGCT

### 2.7 Western blot analysis

Total protein was extracted from KCs or allografts using protein extraction kit (Beyotime, Jiangsu, China). Protein concentrations were detected using a BCA protein quantitative kit (Beyotime, Jiangsu, China). Indicated primary antibodies against Arg‐1, STAT1, p‐STAT1 (CST, USA), IκBα, p‐IκBα, p65, and p‐p65 (Santa Cruz Biotechnology, USA) were used in Western blot following the manufacturer's instructions. GAPDH protein was used as control. Signals were detected through the chemiluminescent reaction using a gel imaging system (ChemiScope 2850, Clinx Science, Shanghai, China).

### 2.8 Construction of AR model of rat OLT with over‐expression of sFGL2

Adeno‐associated virus expressing FGL2 (AAV‐FGL2) and GFP (AAV‐null) were purchased from HanBio (Shang Hai, China). Equivalent dose of AAV‐FGL2 or AAV‐null viral genome (3 × 10^12^ vector genomes/kg) were injected parallel to BN rats 1 month prior to OLT (Lewis to BN) via tail vein. The mRNA levels and serum concentration of sFGL2 were detected by qRT‐PCR and ELISA, respectively.

### 2.9 Morphological changes and function of liver grafts

Allografts were fixed in 10% paraformaldehyde solution, embedded in paraffin blocks, sectioned, and stained with hematoxylin and eosin (H&E) according to standard protocol. Banff criteria were used to score the AR.^28^ The serum was obtained from recipients on day 7 after OLT and used to detect ALT, AST, and TBIL (Jiancheng Bioengineering Institute, China).

### 2.10 Adoptive transfer of KCs

KCs (2 × 10^7^ cells) isolated from allografts of AAV‐FGL2‐treated BN recipient (AAV‐FGL2‐KCs), AAV‐null‐treated BN recipients (AAV‐FGL2‐KCs) on day 7 after transplantation, or liver tissue of Lewis rat (Lew‐KCs) were adoptively transferred to BN recipients via portal vein during OLT (Lewis to BN).

### 2.11 Statistical analysis

SPSS 17.0 software was used for statistical analysis. The results are presented as mean ± SD. The Student's *t* test was used for 2 samples analysis and one‐way ANOVA among different groups. Pearson's correlation analysis was used to evaluate the correlation. Kaplan‐Meier method was used to analyze overall survival. Statistical significance was defined as *P* value <.05.

## 3 RESULTS

### 3.1 sFGL2 is upregulated in rat OLT models of tolerance and positively associated with the frequency of M2 KCs

**Figure 1:**
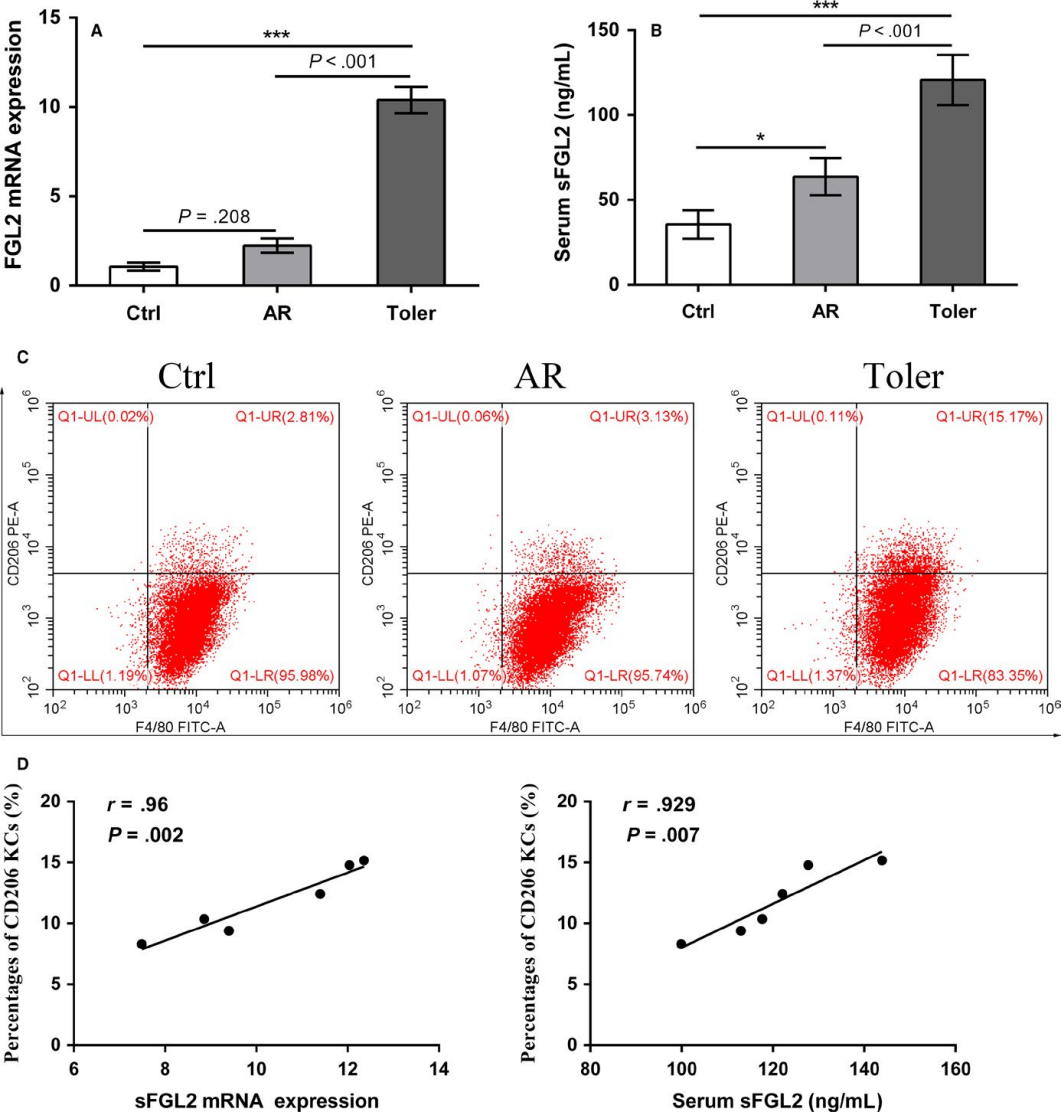
Increased sFGL2 and the frequency of M2 KCs in tolerance group after rat OLT. A, The mRNA levels of FGL2 in liver tissues from BN rats (control group), and that in allografts from recipients in AR or tolerance group were examined by qRT‐PCR. B, Serum levels of sFGL2 from BN rats (control group), recipients in AR or tolerance group were measured by ELISA. C, The F4/80^+^CD206^+^ M2 KCs from BN rats (control group), recipients in AR or tolerance groups were analyzed using flow cytometry. D, Pearson correlation analysis of the frequencies of F4/80^+^CD206^+^ M2 KCs with mRNA and serum levels of sFGL2 in tolerance group. *P < .05, ***P <* *.001 compared with control group. (Ctrl: control group, AR: acute rejection group, Toler: tolerance group)

To investigate the expression profiles of FGL2 in rat liver transplantation, we established rat OLT models of AR and tolerance. qRT‐PCR results showed that FGL2 mRNA levels of allografts were higher in tolerance group than that in AR group. ELISA analysis showed a similar expression pattern (Figure 1A). KCs were then isolated from the grafts to detect the CD206 expression. Flow cytometry analysis showed that the CD206 expression was increased in tolerance group compared with that in AR group (Figure 1B,C). Spearman correlation analysis showed FGL2 expression was positively associated with the frequencies of M2 KCs in rat OLT models of tolerance (Figure 1D).

### 3.2 sFGL2 promotes KCs M2 polarization

**Figure 2:**
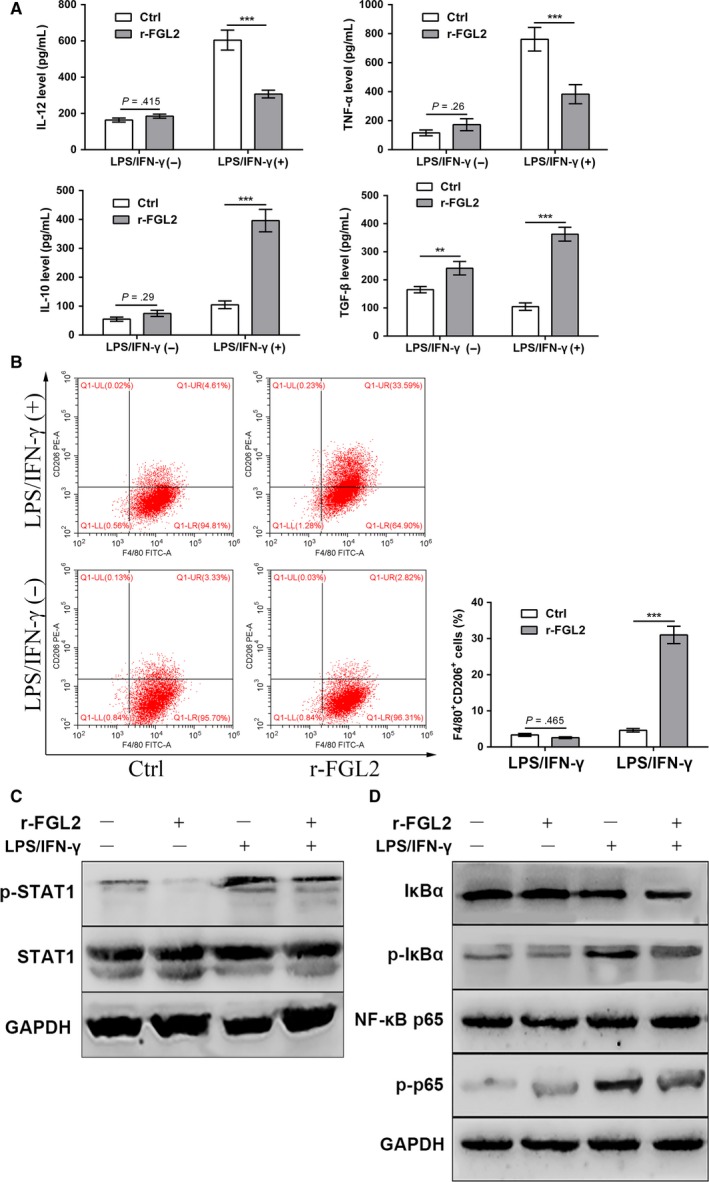
sFGL2 induces KCs M2 polarization and inhibits STAT1 and NF‐κB signaling pathway. A, The supernatant levels of IL‐12, TNF‐α, IL‐10, and TGF‐β in control group and r‐FGL2‐treated group were examined by ELISA. B, The frequencies of F4/80^+^CD206^+^ M2 KCs in control and r‐FGL2‐treated groups were detected by flow cytometry. C, The protein levels of STAT1 and p‐STAT1 in control and r‐FGL2‐treated groups were measured using Western blot. D, The protein levels of IκBα, p‐IκBα, NF‐κB p65, p‐p65 in control and r‐FGL2‐treated groups were measured using Western blot. **P <* *.01, ***P <* *.001 compared to control group. (Ctrl: control group, r‐FGL2: r‐FGL2‐treated group)

sFGL2 has been reported to regulate the polarization of tumor‐associated macrophages in glioma.^24^ We hypothesized that sFGL2 could positively regulate KCs M2 polarization. To verify it, primary KCs were isolated from Lewis rat and treated with LPS/IFN‐γ and r‐FGL2. We found that the productions of IL‐12 and TNF‐α from KCs were increased under LPS/IFN‐γ stimulation, while decreased with the combination of r‐FGL2 and LPS/IFN‐γ. In parallel, the productions of IL‐10 and TGF‐β were increased with the combination of r‐FGL2 and LPS/IFN‐γ (Figure 2A). Flow cytometry assays showed that the CD206 expression levels were higher in KCs with r‐FGL2 and LPS&IFN‐γ treatment compared to those in KCs stimulated by LPS&IFN‐γ (Figure 2B). Together, these data suggested sFGL2 promoted the KCs M2 polarization.

### 3.3 sFGL2 inhibits STAT1 and NF‐κB signaling pathway

It has been reported that STAT1 and NF‐κB signaling pathways are involved in the regulation of macrophage polarization. We hypothesized that sFGL2 could induce KCs M2 polarization via STAT1 and NF‐κB signaling pathways. As expected, Western blot analysis showed that the levels of phosphorylated STAT1, IκBa and NF‐κB p65 were increased in KCs with LPS/IFN‐γ treatment compared to KCs without LPS/IFN‐γ treatment, while the increased expression levels could be attenuated by r‐FGL2 introduction (Figure 2C,D). These results showed that sFGL2 inhibited the STAT1 and NF‐κB pathways in KCs.

### 3.4 sFGL2 inhibits AR of rat OLT

**Figure 3:**
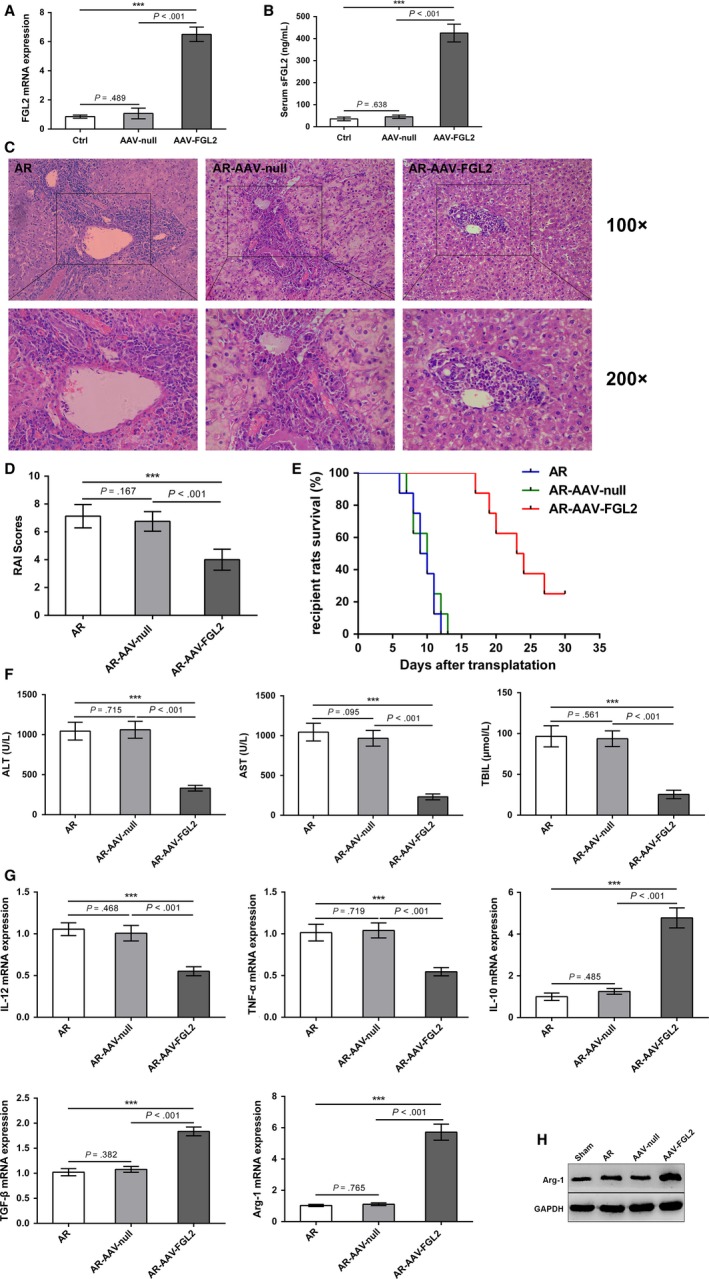
sFGL2 over‐expression in recipients alleviates AR of rat OLT (Lewis to BN). A,B,The mRNA and serum levels of sFGL2 in liver tissues from BN rats (control group), AAV‐FGL2‐treated BN rats or AAV‐null‐treated BN rats were examined using qRT‐PCR or ELISA, respectively. C, H&E staining of allografts on day 7 after OLT. (D) RAI scores were performed to grade AR according to Banff schema. E, Kaplan‐Meier curve was used to perform the survival of recipients. F, Serum levels of ALT, AST, and TBIL on day 7 after OLT. G, The mRNA levels of IL‐12, TNF‐α, IL‐10, TGF‐β and Arg‐1 in KCs isolated from allografts on day 7 after OLT were detected using qRT‐PCR. H, The protein level of Arg‐1 in allografts was measured using Western blot. ***P <* *.001 compared to control group or AR group. (Ctrl: control group, AAV‐FGL2: AAV‐FGL2‐treated BN rats, AAV‐null: AAV‐null‐treated BN rats, AR: saline‐treated BN recipients, AR‐AAV‐FGL2: AAV‐FGL2‐treated BN recipients, AR‐AAV‐null: AAV‐null‐treated BN recipients)

To explore the effect of sFGL2 on AR of rat OLT, we established rat OLT model with recipient receiving AAV‐FGL2 treatment (Figure 3A,B). H&E staining examination for allografts showed less triads (lymphocytes, neutrophils, and eosinophils) infiltration in the portal tracts, minor venous endothelial inflammation and milder bile duct damage in recipients receiving AAV‐FGL2 treatment compared to those in recipients receiving AAV‐null treatment (Figure 3C). We then scored the H&E staining result according to Banff schema. We found that the rejection activity index (RAI) scores of allografts in recipients receiving AAV‐FGL2 treatment were lower than that of allografts in recipients receiving AAV‐null treatment (Figure 3D). Consistently, the overall survival of recipients receiving AAV‐FGL2 treatment was longer than that of recipients receiving AAV‐null treatment (Figure 3E). We also found that the serum levels of ALT, AST, and TBIL were decreased in recipients receiving AAV‐FGL2 treatment compared with those in recipients receiving AAV‐null treatment (Figure 3F).

Moreover, we found that the mRNA levels of IL‐10, TGF‐β, and Arg‐1 in KCs from the allografts of recipients receiving AAV‐FGL2 treatment were higher compared with those in KCs from the allografts of recipients receiving AAV‐null treatment (Figure 3G). Western blot showed a similar expression pattern of Arg‐1 (Figure 3H). These results showed that sFGL2 could inhibit the AR of rat OLT and induce KCs M2 polarization in vivo.

### 3.5 sFGL2 ameliorates AR via inducing KCs M2 polarization

**Figure 4:**
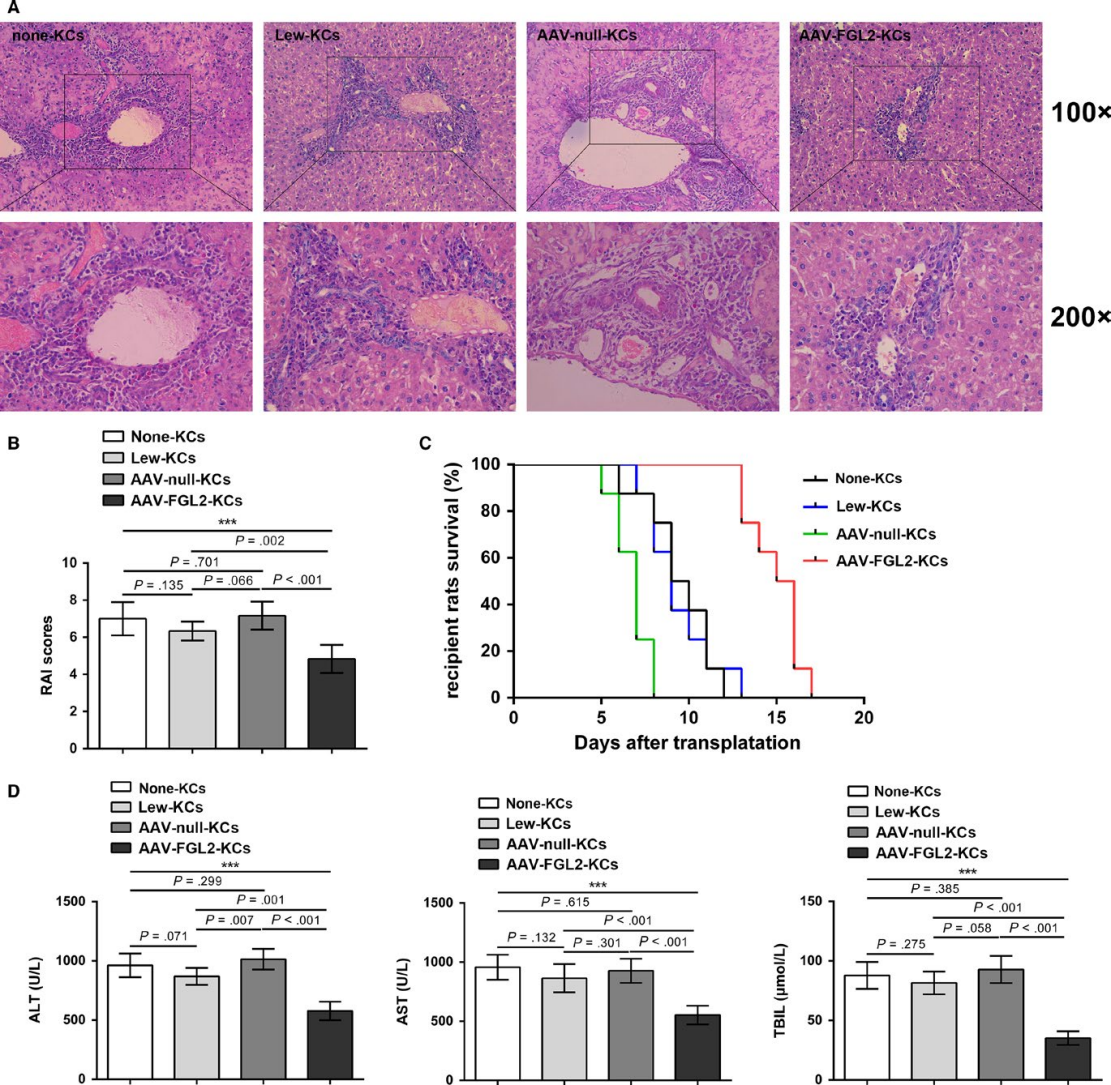
Adoptive transfer of KCs isolated from allografts of AAV‐FGL2‐treated BN recipients ameliorates AR of rat OLT (Lewis to BN). A, H&E staining of allografts on day 7 after OLT. B, RAI scores were performed to grade AR according to Banff schema. C, Kaplan‐Meier curve was used to perform the survival of recipients. D, Serum levels of ALT, AST, and TBIL on day 7 after OLT. ***P <* *.001 compared to AR group. (none‐KCs: OLT with no KCs transferred, Lew‐KCs: OLT with adoptive transfer of KCs from Lewis rats, AAV‐FGL2‐KCs: OLT with adoptive transfer of KCs from AAV‐FGL2‐treated BN recipients, AAV‐null‐KCs: OLT with adoptive transfer of KCs from AAV‐null‐treated BN recipients)

Our previous study showed that sFGL2 could inhibit AR of rat OLT and induce KCs M2 polarization both in vitro and in vivo. We speculated that the KCs M2 polarization was involved in the AR inhibition of rat OLT induced by sFGL2. To examine it, the KCs isolated from the liver tissue of Lewis rat, allograft of recipient receiving AAV‐FGL2 or AAV‐null treatment, were adoptively transferred to OLT rats. H&E staining examinations showed less triads infiltration, minor venous endothelial inflammation, and milder bile duct damage in allografts of recipients receiving AAV‐FGL2‐KCs transfer compared with those in allografts of recipients receiving AAV‐null‐KCs or Lew‐KCs transfer (Figure 4A). The RAI scores of allografts in recipients receiving AAV‐FGL2‐KCs transfer were lower than those of allografts in recipients receiving AAV‐null‐KCs or Lew‐KCs transfer (Figure 4B), and the overall survival of recipients receiving AAV‐FGL2‐KCs transfer was longer than that of recipients receiving AAV‐null‐KCs or Lew‐KCs transfer (Figure 4C). Consistently, the liver function of recipients receiving AAV‐FGL2‐KCs transfer was improved (Figure 4D). These results showed that sFGL2 ameliorated AR by inducing KCs M2 polarization.

## 4 DISCUSSION

In this study, we clarify that sFGL2 induces KCs M2 polarization, and inhibits STAT1 and NF‐κb signaling pathway in KCs. Importantly, we provide the first evidence that sFGL2 ameliorates AR of rat OLT, at least in part, via inducing KCs M2 polarization.

Several studies show that the expressions of FGL2 are upregulated in mouse‐tolerant hepatic and cardiac allografts, and over‐expression of sFGL2 contributes to the acceptance of a fully mismatched heart allograft in mice.^23^, ^29^ Interestingly, other studies show that the serum levels of sFGL2 are higher in patients with AR after renal transplantation compared to patients without AR.^19^, ^30^ These discrepancies might be due to spatiotemporal expression and dynamic changes of sFGL2 in transplantation. Our study showed that both mRNA and serum levels of FGL2 were higher in tolerance group of rat OLT than those in AR group. Importantly, we found that the expression of FGL2 was positively associated with the frequency of M2 KCs, suggesting that sFGL2 may play a suppressive role in AR of rat OLT by regulating KCs M2 polarization.

KCs regulate the immune homeostasis after liver transplantation and KCs M2 polarization contributes to the extension of liver allograft survival.^11^, ^31^ A recent study shows that sFGL2 increases the frequency of glioma‐associated M2 macrophages in mice.^24^ Consistent with this finding, our in vitro results showed that sFGL2 could polarize M1 KCs induced by LPS/IFN‐γ toward the M2 phenotype. Over‐expression of sFGL2 increased the expressions of M2 phenotype‐associated molecules IL‐10, TGF‐β, and Arg‐1 in KCs from allografts while decreased the expression of M1 phenotype‐associated molecules IL‐12 and TNF‐α. These observations reveal that sFGL2 could induce KCs M2 polarization in vivo. Moreover, we found that over‐expression of sFGL2 in recipients could ameliorate AR of rat OLT. Importantly, we found that adoptive transfer of AAV‐FGL2‐KCs to recipients inhibited AR, indicating that sFGL2 inhibited AR via inducing KCs M2 polarization. M2 KCs could produce TGF‐β and IL‐10,^5^, ^32^ which mediates immune tolerance in mice liver injury by down‐regulating the production of TNF‐α and IL‐12.^12^, ^33^ In addition, KCs M2 polarization contributes to the apoptosis of M1 KCs in fatty liver disease.^34^ These findings support our results that sFGL2 plays a protective role in AR of rat OLT via inducing KCs M2 polarization.

Although extensive studies have demonstrated that sFGL2 acts on macrophages through the 2 special receptors, FcγRIIb and FcγRIII, the downstream signaling pathway responsible for the polarization of KCs is unclear.^35^, ^36^ FcγRIIb is decreased by the TLR4 agonists, in contrast, up‐regulated by IL‐4 and IL‐10.^37^, ^38^, ^39^ Moreover, the suppression of immune globulin on macrophages via inhibiting STAT1 signaling pathway is FcγRIII dependent.^40^ It has been shown that Tim‐3 induces M2 macrophage polarization via inhibiting STAT1‐miR‐155‐SOCS1 pathway.^41^ Based on these findings, we speculated that sFGL2 induced KCs M2 polarization by inhibiting STAT1 and NF‐κB pathway. As expected, we found that r‐FGL2 significantly inhibited the phosphorylation of STAT1 and NF‐κB induced by LPS/IFN‐γ. However, the exact function of Fcγ receptors on KCs and detailed signaling pathway needs to be further investigated.

Taken together, we revealed that sFGL2 induced KCs M2 polarization both in vitro and in vivo. We found sFGL2 could suppress the STAT1 and NF‐κB signaling pathway, which might contribute to KCs M2 polarization. Importantly, we revealed that both over‐expression of sFGL2 in recipients and adoptive transfer of KCs from sFGL2 over‐expression allografts contributed to alleviating AR of rat OLT. Our results might provide a new preventive and therapeutic strategy to manage patients with AR after liver transplantation.

## 5 ETHICS STATEMENT

The animals involved in this study were performed strictly according to the guidelines approved by the Animal Ethics Review Committee of Chongqing Medical University. All the surgeries were performed under diethyl ether inhalation anesthesia.

## CONFLICT OF INTEREST

We declare no conflict of interest.
