# Supplementary figures and images for: Soluble fibrinogen‐like protein 2 ameliorates acute rejection of liver transplantation in rat via inducing Kupffer cells M2 polarization
Source: Cancer Med. 2018 May 10;7(7):3168–77. doi: 10.1002/cam4.1528 (PMC6051168; doi:10.1002/cam4.1528)

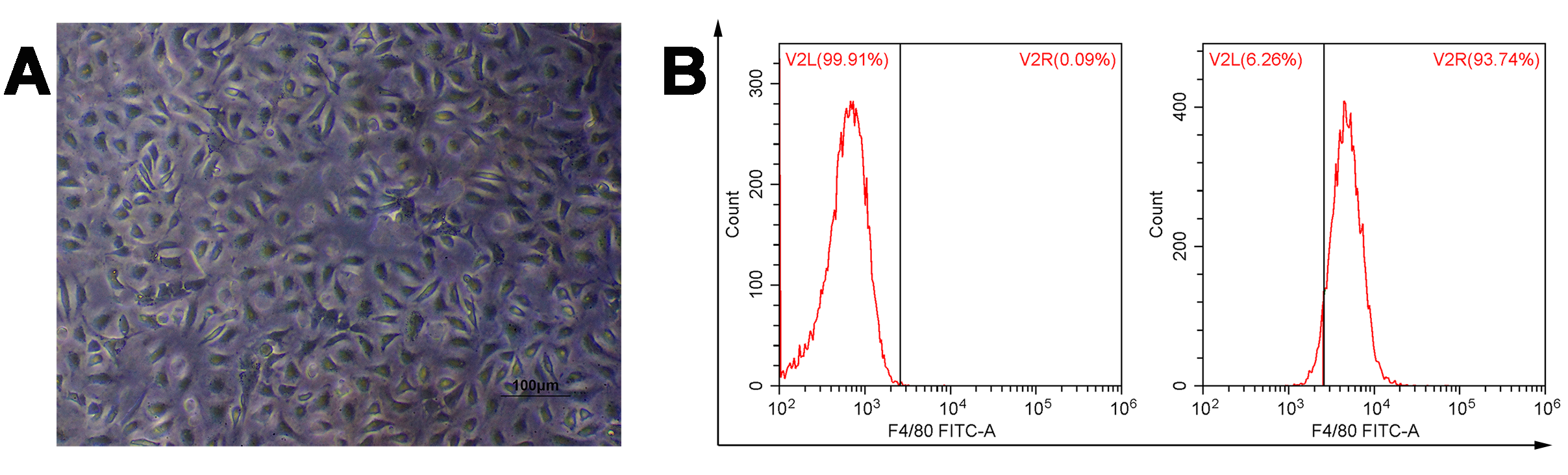

Supplement: Supplementary file 1 [file CAM4-7-3168-s001.tif]

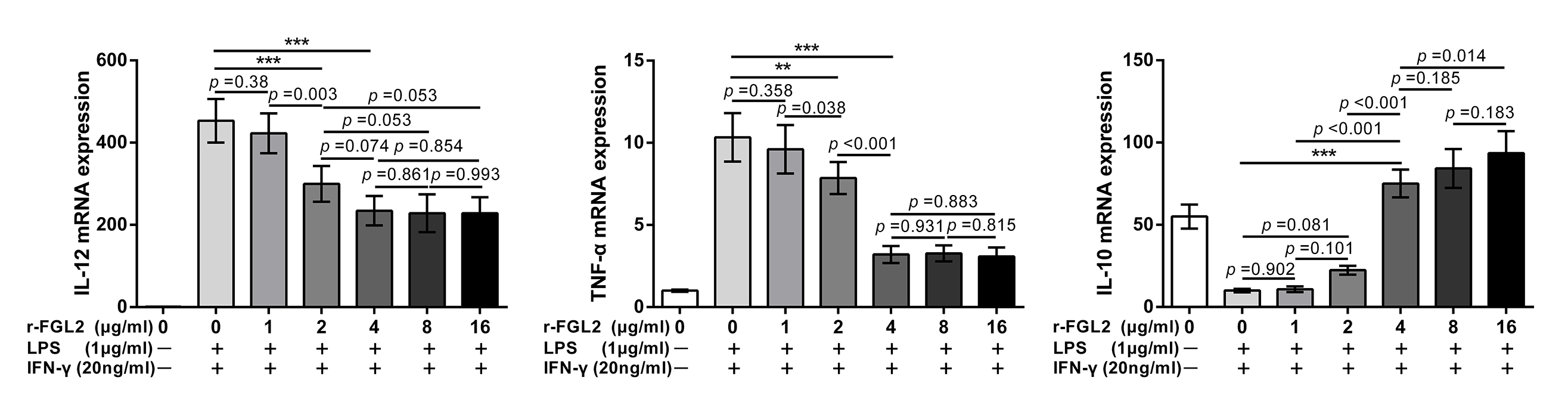

Supplement: Supplementary file 2 [file CAM4-7-3168-s002.tif]
