# Supplementary material for: Soluble fibrinogen‐like protein 2 ameliorates acute rejection of liver transplantation in rat via inducing Kupffer cells M2 polarization
Source: Cancer Med. 2018 May 10;7(7):3168–77. doi: 10.1002/cam4.1528 (PMC6051168; doi:10.1002/cam4.1528)
Supplement: Supplementary file 3 [file CAM4-7-3168-s003.doc]

**Supplementary files**

**Supplementary Figure 1. KCs cultured for 48h.** (A) KCs were isolated from rat liver tissues and cultured for 48h (magnification ×200). (B) The purity of F4/80+ KCs was greater than 90% by flow cytometry analysis.

**Supplementary Figure 2. sFGL2 acted on KCs stimulated by LPS/IFN-γ in a dose-dependent manner.** LPS (1μg/ml) and IFN-γ (20ng/ml) stimulated KCs in the presence of graded concentrations of r-FGL2 for 24h, the mRNA levels of IL-10, TNF-α and IL-12 in KCs were determined by qRT-PCR analysis. **p* <0.05, ****p* <0.001 compared to LPS/IFN-γ stimulated group.
